# Stress Effect in the Knee Joint Based on the Fibular Osteotomy Level and Varus Deformity: A Finite Element Analysis Study

**DOI:** 10.3390/bioengineering10091003

**Published:** 2023-08-24

**Authors:** Yeokyung Kang, Jungsung Kim, Jae Ang Sim, Myeong Moon, Jong-Chul Park, Sung Ha Cho, Byung Hoon Lee

**Affiliations:** 1 Central Research & Development Center, Corentec Company Co., Ltd., 33-2, Banpo-daero 20-gil, Seocho-gu, Seoul 06649, Republic of Korea; ykkang@corentec.com (Y.K.); jskim@corentec.com (J.K.); 2 Department of Orthopedic Surgery, Gachon University College of Medicine, Namdong-gu, Incheon 21565, Republic of Korea; sim_ja@gilhospital.com (J.A.S.); general225@gilhospital.com (S.H.C.); 3 Medical School, Gachon University College of Medicine, Namdong-gu, Incheon 21565, Republic of Korea; river0305@naver.com; 4 Cellbiocontrol Laboratory, Department of Medical Engineering, Yonsei University College of Medicine, Seoul 03722, Republic of Korea; parkjc@yuhs.ac

**Keywords:** fibular osteotomy, varus angle, finite element method, peak von Mises stress, meniscus, cartilage stress

## Abstract

Proximal fibular osteotomy (PFO) was found to relieve pain and improve knee function in patients with medial compartment knee osteoarthritis (OA). Therapy redistributes the load applied from the inside to the outside and alleviates the load applied on the inside through fibula osteotomy. Therefore, the clinical effect of fibular osteotomy using the finite element (FE) method was evaluated to calculate the exact change in stress inside a knee joint with varus deformity. Using CT and MRI images of a patient’s lower extremities, 3D models of the bone, cartilage, meniscus, and ligaments were constructed. The varus angle, representing the inward angulation of the knee, was increased by applying a force ratio in the medial and lateral directions. The results showed that performing proximal fibular osteotomy led to a significant reduction in stress in the medial direction of the meniscus and cartilage. The stress reduction in the lateral direction was relatively minor. In conclusion, the study demonstrated that proximal fibular osteotomy effectively relieves stress and redistributes the load in the knee joints of patients with medial compartment knee osteoarthritis. The findings emphasize the importance of considering force distribution and the position of fibular osteotomy to achieve optimal clinical outcomes.

## 1. Introduction

Varus alignment of the lower extremities is associated with a higher load on the medial compartment of the tibiofemoral joint during weight bearing, and increasing varus alignment contributes to increasing varus osteoarthritis (OA) [1,2,3,4,5]. High tibial osteotomy (HTO) is generally accepted as one of the most useful surgical procedures for treating medial compartment osteoarthritis of the knee, and it incurs less degenerative changes in the medial compartment [2]. HTO decreases the moment arm of the ground reaction force and further offloads the medial compartment of the knee by changing the knee alignment from varus to valgus [6].

However, alignment correction procedures such as HTO for patients with neutral or mild varus knee alignment may be excessive and can result in inferior outcomes with postoperative valgus alignment [7]. Recently, proximal fibular osteotomy (PFO) was found to relieve pain and improve knee function in patients with medial compartment knee OA. Also, it is a unique way to treat knee osteoarthritis, which to date cannot be fully explained by biomechanical studies [8]. The treatment effect on medial knee OA can be predicted, as the medial load is reduced without actually correcting the alignment of the knee joint. This redistributes the load applied from the inside to the outside and alleviates the load applied on the inside through fibula osteotomy [9]. However, there is still widespread controversy, because most studies lack sufficient quantitative evidence regarding changes in the biomechanical properties of the knee in response to fibular osteotomy (OT).

Therefore, in this study, the clinical effect of fibular osteotomy using the finite element (FE) method was evaluated to calculate the change in stress inside a knee joint with varus deformity. In addition, we sought to establish a clinical basis by identifying the surgical effect according to the position of the fibular osteotomy and the most appropriate level of osteotomy. Furthermore, this procedure does not require additional surgical intervention for fixative removal such as screws or metal plates, and immediate weight bearing is possible after surgery [10]. Several authors have reported that fibular OT could improve both the radiographic appearance and function of varus knees with osteoarthritis [8,11].

However, the precise mechanism explaining medial compartment decompression and the deloading effect of fibula osteotomy has not yet been confirmed. Fibula osteotomy still requires an exact theoretical mechanism background, including loading condition and measured material properties of the bone and metallic implants; thus, hard evidence is necessary to determine the influence of medial compartment deloading. Femoral cartilage, tibial cartilage, and meniscus were selected for confirmation of evidence. The knee joint is driven by contact between the femur and tibia and the main components responsible for this joint motion are the femoral cartilage, tibial cartilage, and meniscus. Predicting and preventing the occurrence of arthritis depends on the magnitude and distribution of stress in these three components. Therefore, to ensure the clinical validity of this study and enable its application in treatment decisions, we aimed to determine the stress levels occurring in these three components.

Finite element analysis (FEA) is a highly reliable method that simulates various consequences without clinical trials, and numerous biomechanical studies have used this method to calculate the internal stress. Because FEA can be tested by reflecting the number of cases that can occur in actual clinical practice, it is possible to perform the above-mentioned theoretical mechanism under optimal conditions [12,13]. Therefore, in this study, the clinical effect of fibula osteotomy was evaluated using FEA to calculate the exact stress changes in an internal knee joint with a varus deformity.

To the best of our knowledge, no studies have presented a theoretical basis for an appropriate level of OT when performing fibular OT, and the difference in the effect of reducing internal load according to the level of OT is also unknown. Therefore, the aim of this study is to investigate the clinical deloading effect of the medial compartment and the surgical effect about the degree of fibular osteotomy, based on the analysis of the results of varus deformity single load according to the single level of PFO in previous studies. We also confirmed the effect of fibular osteotomy (FO) concerning the severity of varus deformity, including changes in load at different varus deformity levels, as well as the correlation between the FO level and varus deformity. These findings will establish a clinical basis for determining the appropriate level of fibular osteotomy during surgery.

## 2. Materials and Methods

### 2.1. Construction of a Bone Model Based on Patient Image Data

**Figure 1:**
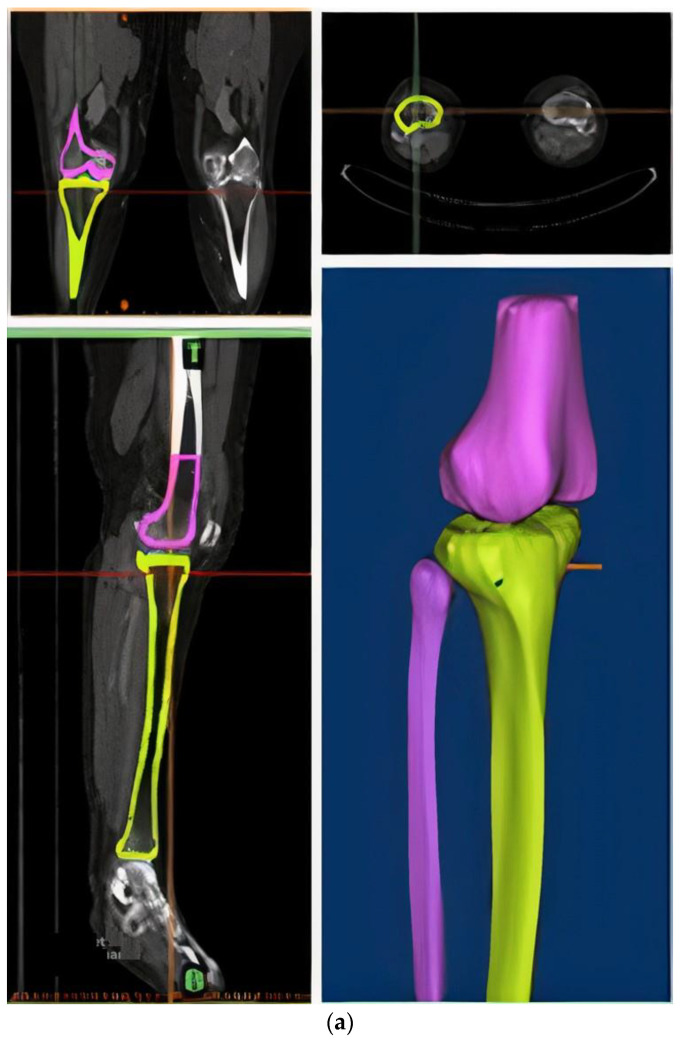

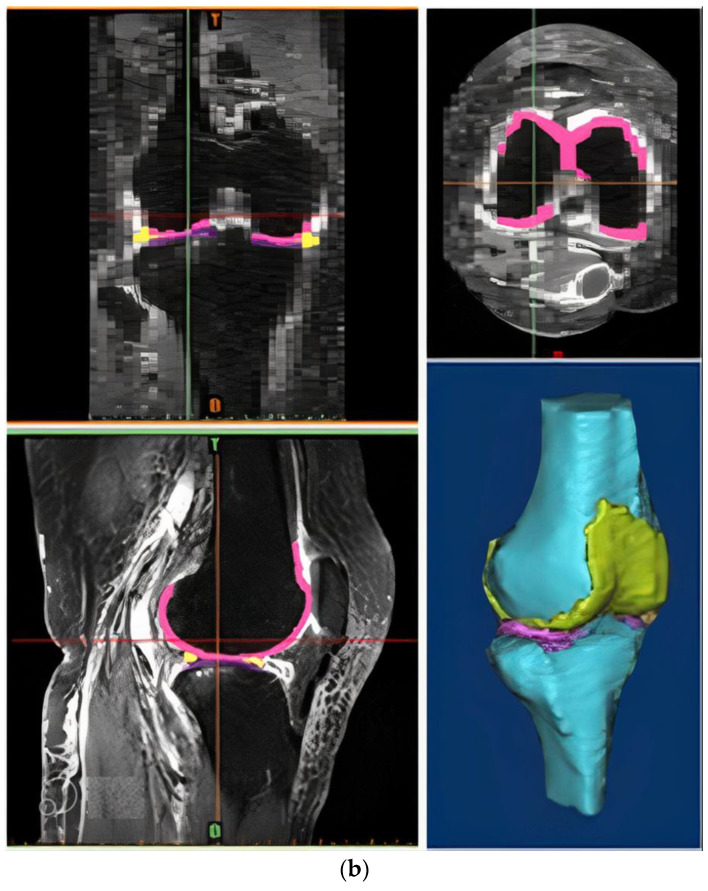
Data reconstruction in Mimics software (**a**) CT data (**b**) MRI data.

**Figure 2:**
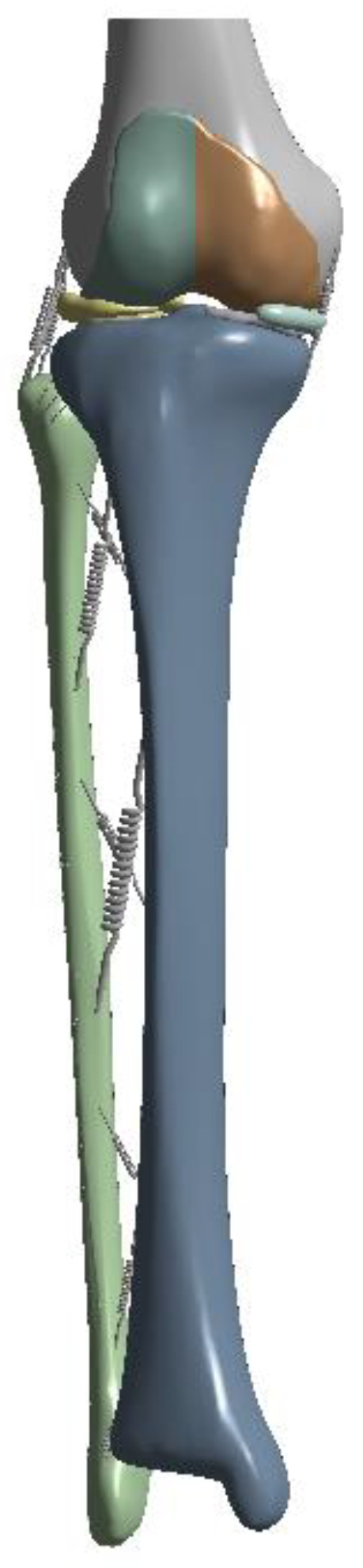
Intact bone model.

In this study, the radiology image data of a 45-year-old Korean woman (height: 152.8 cm; weight: 73.4 kg) were used to construct a bone model. CT data of the right lower limb in intact condition and MRI data of the right knee joint were used. The image data in Digital Imaging and Communications in Medicine (DICOM) format were used to reconstruct the model using Mimics 16.0 (Materialize NV, Leuven, Belgium) software. The distal part of the femur and the entire tibia and fibula were obtained as 3D data from the CT data (Figure 1a), and the distal femur, proximal tibia, femoral cartilage, tibial cartilage, and meniscus were obtained as 3D data from the MRI data (Figure 1b). The reconstructed 3D models were combined using the global registration of 3-Matic 8.0 (Materialize NV, Belgium). The distal femur and proximal tibia obtained from the MRI data were moved and aligned based on the tibia and femur bones from the CT data, and the cartilage and meniscus from the MRI data were moved along with the bone-aligned model. Finally, as shown in Figure 2, an intact bone model was constructed using the distal femur, tibia, and fibula from the CT data and the cartilage and meniscus from the MRI data.

**Figure 3:**
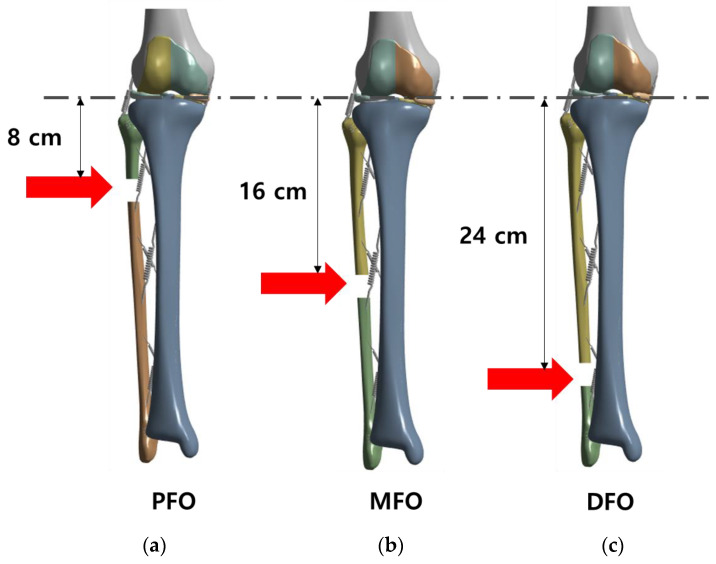
Fibula osteotomy position: (**a**) Proximal fibula osteotomy (PFO), (**b**) Middle fibula osteotomy (MFO), (**c**) Distal fibula osteotomy (DFO).

For comparison between the intact model and the osteotomy model, the intact model was implemented as a fibula osteotomy model by cutting a certain position of the fibula, as shown in Figure 3. Osteotomy was performed on the 8 cm from the lateral tibia plateau on the lower end at a 2 cm distance for proximal fibula osteotomy (PFO, Figure 3a), on the 16 cm from the lateral tibia plateau on the lower end for middle fibula osteotomy (MFO, Figure 3b), and on the 24 cm from the lateral tibia plateau on the lower end for distal fibula osteotomy (DFO, Figure 3c) [13].

The intact, PFO, MFO, and DFO models were remeshed in Ansys 2022R2 (Ansys, Inc., Canonsburg, PA, USA) software, and the numbers of elements and nodes of each model are listed in Table 1.

### 2.2. Establishment of Ligament

**Figure 4:**
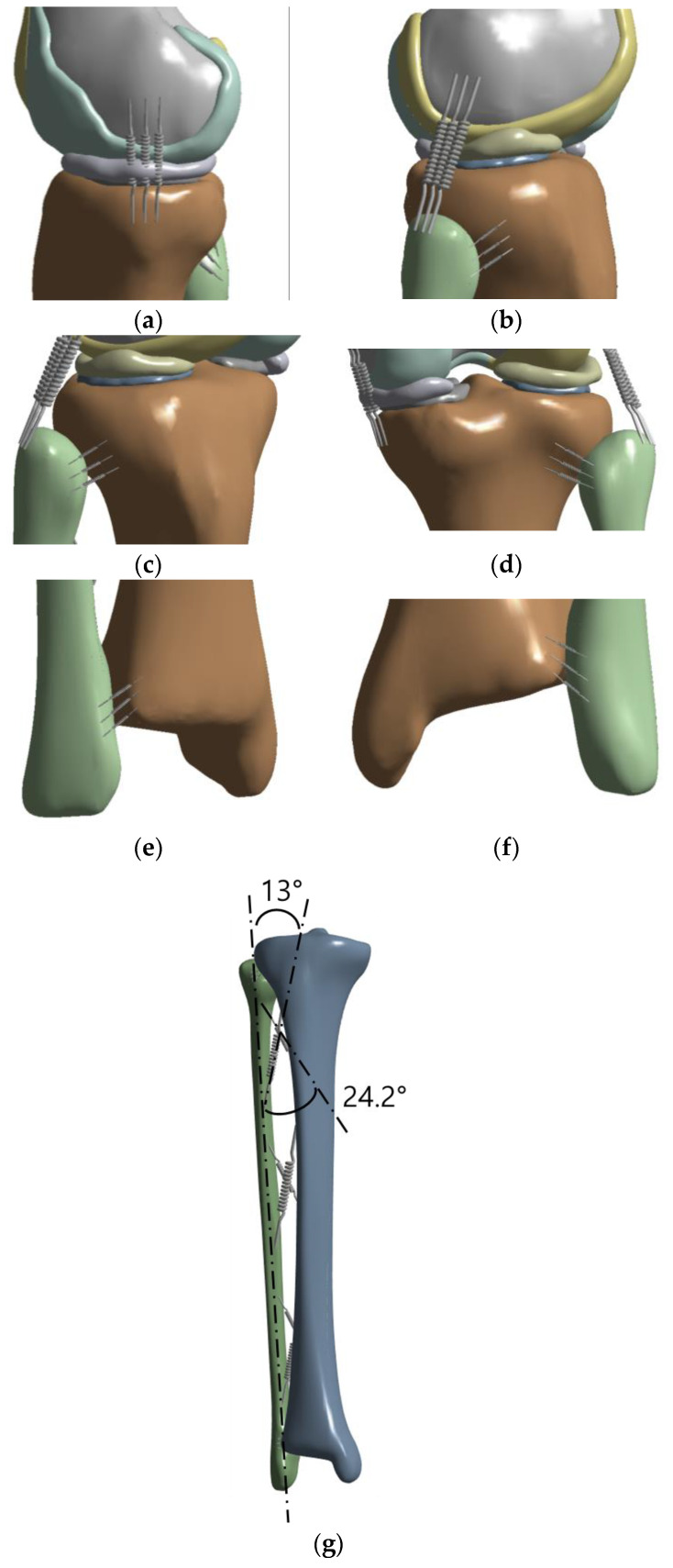
Ligament: (**a**) MCL, (**b**) LCL, (**c**) Proximal anterior tibiofibula ligament, (**d**) Proximal posterior tibiofibula ligament, (**e**) Distal anterior tibiofibula ligament, (**f**) Distal posterior tibiofibula ligament, (**g**) Interosseous membrane.

To ensure that the bone model reflected the real-world situation as accurately as possible and to strengthen the constraints for analysis between the femur and tibia and between the tibia and fibula, the ligament was built in the form of a spring in the bone model (Figure 4). The position of the ligament was designated based on data from radiological images and previous studies [14,15], and one type of ligament was divided into three springs and fixed. As shown in Figure 4a,b, the medial collateral ligament (MCL) and lateral collateral ligament (LCL) were established, and as shown in Figure 4c–f, the proximal and distal anterior and posterior tibiofibular ligaments were constructed. As shown in Figure 4g, the interosseous membrane was divided into two types and fixed anteriorly and posteriorly; the anterior interosseous membrane was fixed at an angle of 13° and the posterior interosseous membrane at an angle of 24.2° based on the fibula axis [16].

### 2.3. Material Properties

In this study, the material properties were applied as shown in Table 2. For the properties of cortical bone, anisotropy properties were applied, and for cancellous bone, a Young’s modulus of 1061 MPa and Poisson’s ratio of 0.225 were used. For cartilage, a Young’s modulus of 12 MPa and Poisson’s ratio of 0.45 were applied, and for the meniscus a Young’s modulus of 80 MPa and Poisson’s ratio of 0.3 were applied. The ligament stiffness applied per spring for each part was determined. A load was not imposed on the ligament during compression, and stiffness was applied only during tension.

### 2.4. Load and Boundary Conditions

The load and boundary conditions were set using Ansys 2022R2 software (Ansys, Inc., Canonsburg, PA, USA). The contact conditions are presented in Table 3. The tibia and fibula were completely fixed in contact with the distal talus, and the cortical and cancellous bones were also completely fixed. In addition, the femur–cartilage and tibia–cartilage–meniscus were completely fixed, and a friction coefficient of 0.2 was assigned between the femoral cartilage and meniscus, and between the femoral cartilage and tibial cartilage, which move by friction at the knee joint. The contact between the tibia cortical bone and fibula cortical bone had a friction coefficient of 0, but no separation was applied [11,23,24].

The weight of the model was confirmed to be 73.5 kg, and “body weight (kg) × 10 (m/s^2^)” was applied as a vertical load of 720 N. This load was applied at the proximal metaphysis where the femur was cut. [11] In order to confirm the influence of varus on the important contact elements of the model together, the same load of 720 N was applied to each of the medial and lateral parts of the proximal tibia as “Intact, 2:1, 3:1, 4:1”. The ratio represents the magnitude of the load along the medial and lateral directions in the three loading conditions, excluding the intact model. The ratio is the result of dividing by the total load, and it was confirmed that the load is applied at a ratio of 2:1 (480 N Medial:240 N Lateral) to the medial and lateral condyles in normal alignment. A medial-lateral condylar load ratio of 3:1 (540 N medial:180 N lateral) or 4:1 (576 N medial:144 N lateral) was chosen to assume a varus knee joint. In other words, we intended to determine whether analyzing patients with severe varus by classifying them by load had a significant effect on the stress change of important elements of the model. Accordingly, 12 types of analysis models were built (Table 4). Under the above boundary conditions, the stresses of meniscus, tibial cartilage, and femoral cartilage of intact model and PFO-MFO-DFO model are reported.

### 2.5. Validation of the Bone Model Using a Finite Element Analysis

In this study, the convergence of a finite element model was evaluated using the error rate based on the interpretation of the mesh factor number. We aimed to evaluate the accuracy of worst-case specifications and standard model efficacy using the convergence item in the Ansys software program. For the evaluation, we used the “convergence” function with an “adaptive mesh refinement” approach that involved repeated interpretations with increasing mesh factors. In the repeated interpretation process, we used the ratio of factors that slowly increased with the “refinement depth 1/2” setting and an acceptable percentage of chance for convergence set at 5% to evaluate each finite element model’s errors [25].

## 3. Results

**Figure 5:**
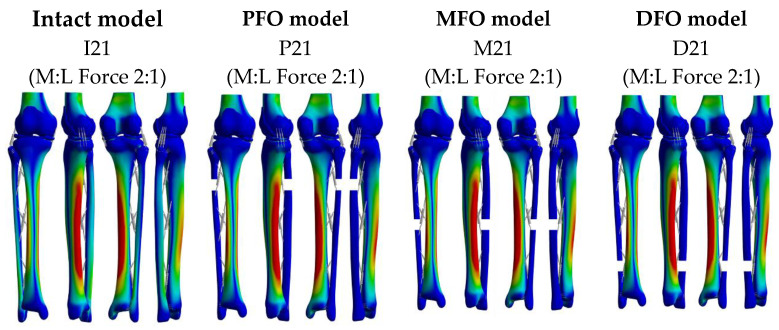

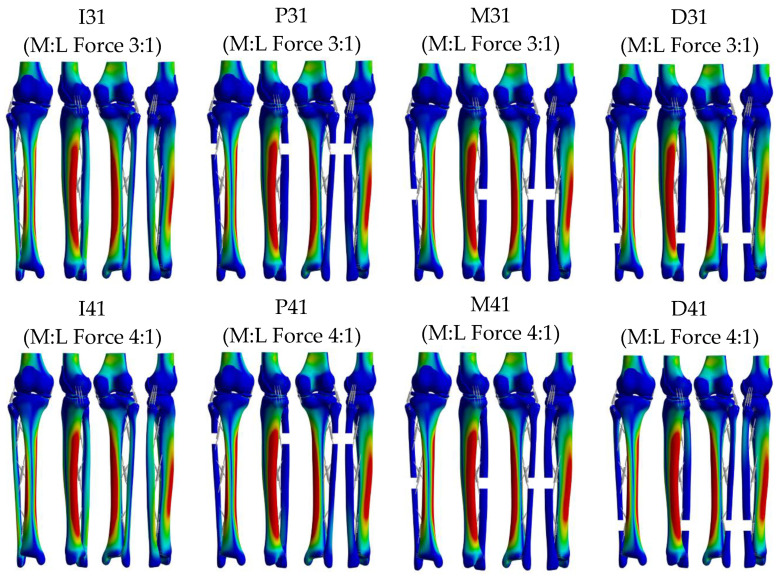
Stress distribution in the whole body.

The von Mises stress distributions for the 12 models are shown in Figure 5. Peak stress occurred in the medial tibial shaft in all models. The peak von Mises stress of the meniscus, tibial cartilage, and femoral cartilage were derived to confirm the change in stress in the medial and lateral directions according to the degree of fibula osteotomy and varus.

**Figure 6:**
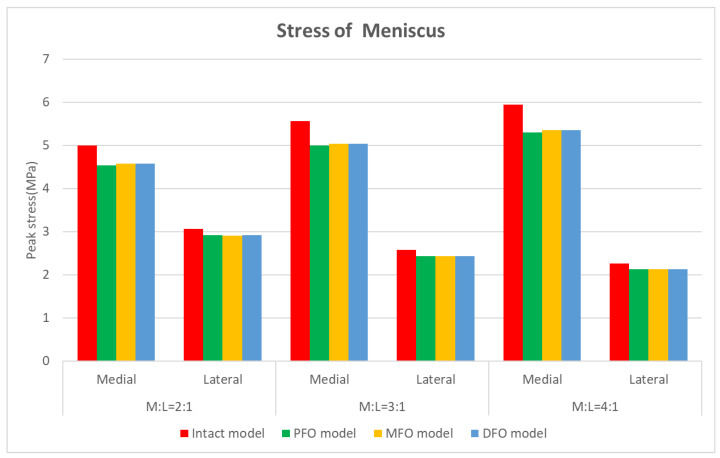
Graph showing meniscus stress.

**Figure 7:**
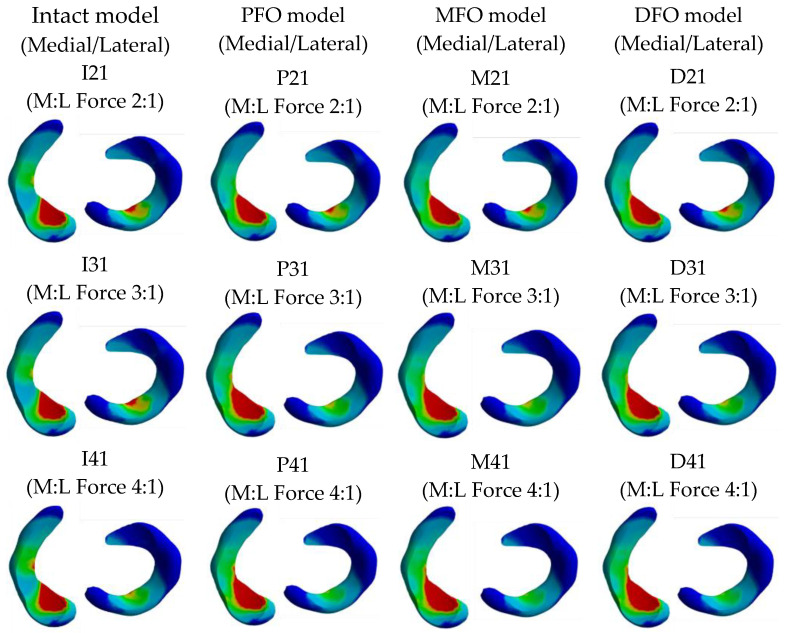
Stress distribution in the meniscus.

The stress results derived from the meniscus are shown in Table 5 and Figure 6 and Figure 7.

**Figure 8:**
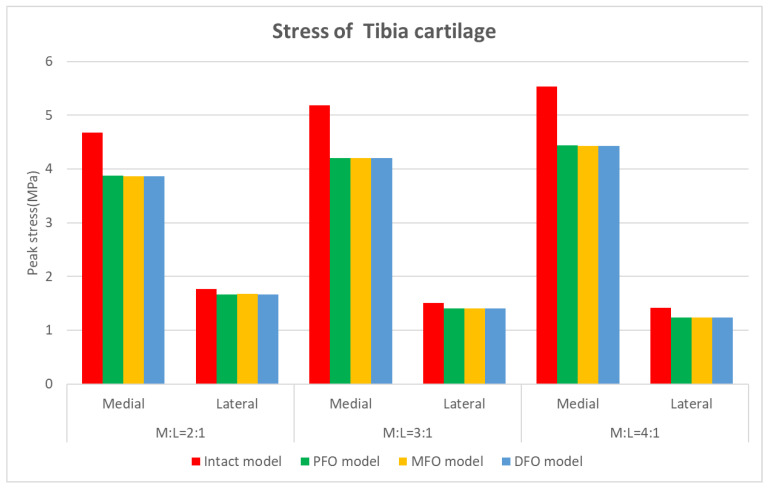
Graph showing tibial cartilage stress.

**Figure 9:**
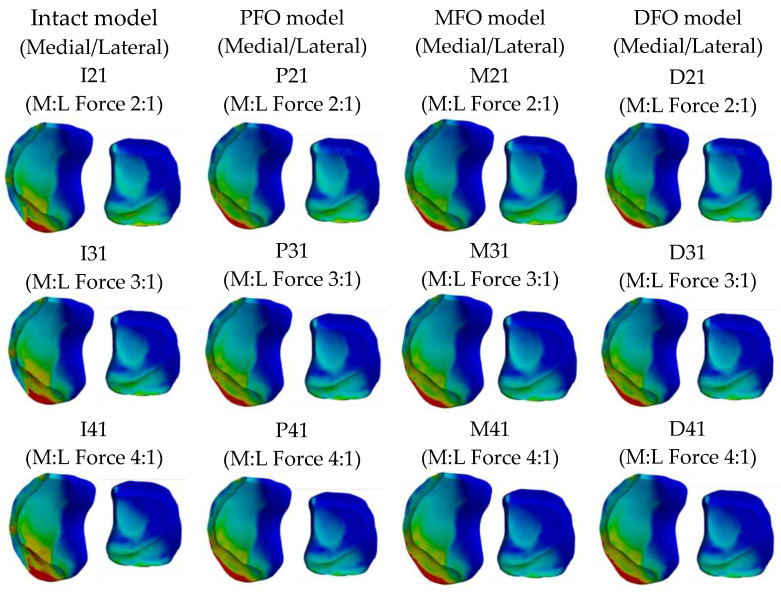
Stress distribution in the tibial cartilage.

The stress results derived from the tibial cartilage are shown in Table 6 and Figure 8 and Figure 9. In Meniscus and tibial cartilage, the peak von Mises stress was reduced overall in the FO model compared to the intact model. The stress in the medial direction was higher than that in the lateral direction, and it was confirmed that the stress in the medial direction significantly decreased as FO implementation. In addition, the lateral direction stress decreased slightly after fibula osteotomy. Regarding the analysis of force changes in the medial and lateral directions, as the varus angle increased (medial force increases), the amount of reduction in medial direction stress showed a tendency to increase after fibula osteotomy was performed.

**Figure 10:**
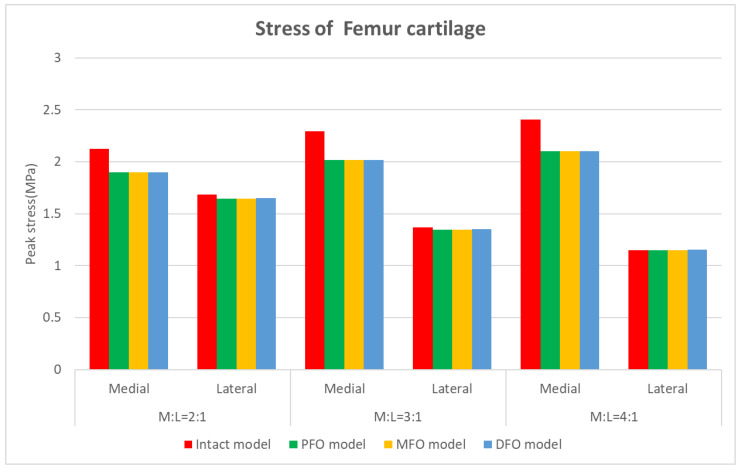
Graph showing stress in the femoral cartilage.

**Figure 11:**
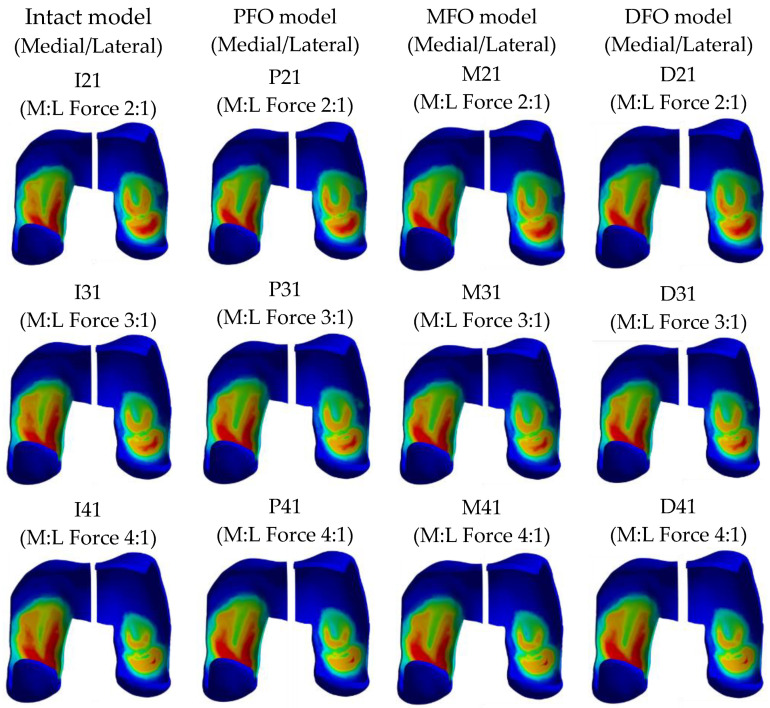
Stress distribution in the femoral cartilage (superior view).

**Figure 12:**
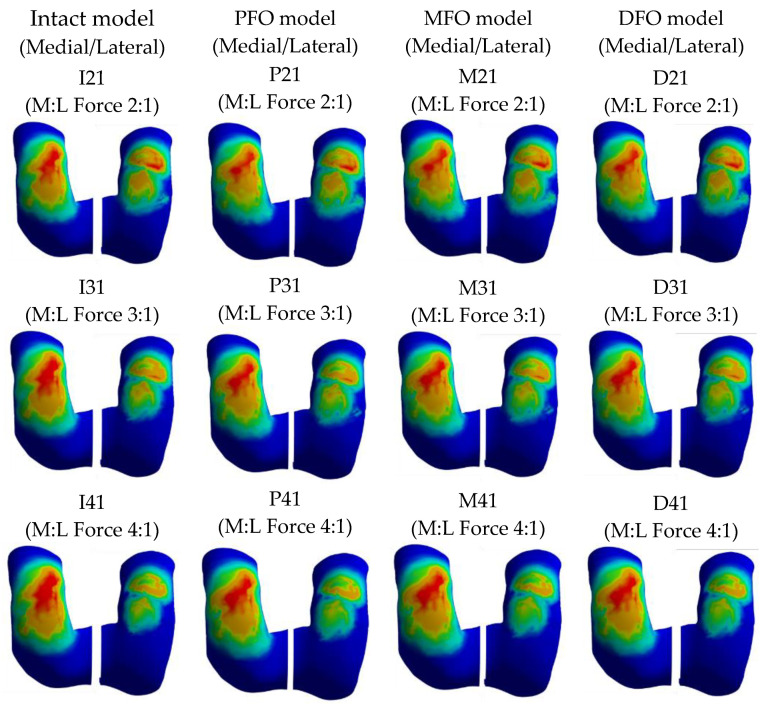
Stress distribution in the femoral cartilage (inferior view).

The stress results derived from the femur cartilage are presented in Table 7 and Figure 10, Figure 11 and Figure 12. As for femoral cartilage, the peak von Mises stress overall decreased in the FO model compared to the intact model, and the stress in the medial direction was higher than that in the lateral direction, and it was confirmed that the stress in the medial direction significantly decreased as FO was implemented. In addition, stress in the lateral direction also slightly decreased after fibula osteotomy, but at a medial-lateral force ratio of 4:1, the reduction rate was numerically less than 1.5%. In the analysis result according to medial and lateral force change (alignment change), as the varus angle increases (medial force increases), there is a certain tendency that the amount of stress reduction in the medial direction increases after fibula osteotomy.

## 4. Discussion

The principal findings of this study are that, following fibula osteotomy, the stress loaded on the knee joint was relieved in general, especially in the medial compartment, and there were no differences in stress change according to the fibula osteotomy level. These findings support other findings on fibula osteotomy in patients with knee OA [9,11,15,23]. Thus, in 2015, fibula osteotomy was described as an alternative surgical method instead of HTO, considering its small incision, short surgery time, and the clinical effect of medial compartment decompression [8,24].

While evaluating the clinical effect of fibula osteotomy, we observed maximum stress changes in the femoral cartilage, tibial cartilage, and meniscus. The probability of occurrence of arthritis can be predicted and prevented depending on the magnitude and distribution of stress in the three components. Therefore, in order to ensure the clinical validity of the results of this study and to be able to refer to them when selecting the direction of treatment in the clinic through the results, we tried to confirm how much stress occurs in the three components. After fibula osteotomy, the maximum stress in the femoral cartilage, meniscus, and tibial cartilage on the medial side markedly decreased by approximately 10.7%, 8.4%, and 17.4%, respectively. Those in the lateral side decreased slightly by approximately 2.3%, 4.7%, and 5.7%, respectively. Nie et al. revealed that after fibula osteotomy, the stress on the medial side is transferred to the posterolateral side of the knee joint [26], and the results of this study can also predict the same stress transfer. However, some studies have shown that the stress in the distal tibial cartilage has increased [24]. Our study showed that stress decreased mainly on the medial side and was also relieved on the lateral side. Thus, patients with OA experience medial pain relief and improvement in ambulation when the stress in the medial compartment decreases [23].

Considering the effect of the fibula osteotomy level, neurovascular components should be considered in the surgical section. The common peroneal nerve should be considered in the proximal part of the fibula. In the middle part of the fibula, the peroneal vessels and nerves undergo neurovascular damage during mid-shaft fibula osteotomy. At the distal part of the fibula, most vessels are present and must be protected [27]. However, we found no differences in the level of fibular osteotomy. Therefore, it is possible to select the level of surgery considering the effectiveness of the procedure and difficulty of surgery performing according to the patient’s condition.

This study was performed using FEA and not a clinical method. However, various methods have been used to reduce the gap between our study and the real-world clinical settings. By designing the bone model for the FEA study, the boundary conditions between the bone, cartilage, and meniscus were mostly bonded, and only the boundary condition between the tibia and fibula bones was set up as “no separation” [11,24,26]. Ligaments were made in an anatomical position to maintain appropriate tension [16,20,21,22]. To verify the finite element model used for assessing the effectiveness of the variable model for each specification, we performed convergence evaluation, in which we assessed the change in peak von Mises stress with an increase in the number of elements. The error rate of peak von Mises stress change for each model in adaptive mesh refinement convergence was confirmed to be 0.36–4.57%. Therefore, this confirms that the maximum error rate for each model element is within 5%. However, not all references are reliable, as all studies have inherent limitations. Despite this, unlike other in studies, in the current study the tibia and fibula were implemented in a single bone model. In addition, the proximal anterior and posterior tibiofibular ligaments were made by setting three springs, and the interosseous membrane was made using six springs according to anatomical studies. There was a significant difference considering the presence of the fibula; thus, our study suggests that implementing fibula bone is necessary in lower limb FEA studies.

Nevertheless, there are some limitations to FEA research, such as difficulties in simulating a real-world clinical setting [28]. And there is a limitation in that the results were compared only with the peak von Mises stress, but the purpose of the study was not to check various stress values according to the change of conditions. Centering on the peak von Mises stress, when the varus load is the same, the stress change according to the level of FO was confirmed, and also, when the FO level is the same, the change in the result according to the change of the varus load was confirmed. Therefore, the purpose of this study was to find the optimal level of FO according to the severity of varus patients. If the comparison was made by including the mean value, median value, and quartiles value in addition to the peak von Mises stress value, more correlations could be identified. However, since there were 12 types of boundary condition variables including load and model shape in this study, the analysis range of the results was limited to the peak von Mises stress only. Furthermore, it is important to note that this study differs from clinical trials and analyses of dynamic motion, as it focused on static posture simulation using FEA. This is a limitation because statistical validity does not exist as a bone model was created using information from a single patient and single leg stance. However, as mentioned earlier, even though we used FEA, the model was implemented based on anatomical knowledge, and a visible confirmation of postoperative knee compartment stress change was possible. In addition, even if the diagnosis of each patient is different, because the correlation of FO according to the severity of varus patients was analyzed, the results were schematized, and the trend was confirmed. Thus, this study has sufficient clinical relevance and helped us determine the clinical effect of fibula osteotomy.

## 5. Conclusions

A comparison of the peak von Mises stress results between the intact model of 2:1 force ratio and the PFO model of 2:1 force ratio, MFO model of 2:1 force ratio, and DFO model of 2:1 force ratio that underwent fibula osteotomy shows that the meniscal and cartilage stress in the medial direction significantly decreased, while that in the lateral direction tended to decrease slightly. In particular, compared with the intact model of 2:1 force ratio, the stress in the medial tibial cartilage decreased from 17.3% to 17.4% in the PFO model of 2:1 force ratio, MFO model of 2:1 force ratio, and DFO model of 2:1 force ratio, while the stress in the lateral femoral cartilage decreased from 5.6% to 5.8%, showing a difference in the amount of change in the medial and lateral directions. When the force was increased on the medial compartment before OT, the stress decreased more markedly during fibula osteotomy. That is, it was confirmed that the stress of meniscus, tibial cartilage, and femoral cartilage was significantly reduced in the medial direction, and the effectiveness of fibula osteotomy increased as the varus angle was more severe. In addition, given that there is no difference in FO level, it is recommended to choose an osteotomy level that is convenient for the surgeon.

## Figures and Tables

**Table 1:** The number of nodes and elements in each model.

Variable	Number of Nodes	Number of Elements
Intact model	668,372	444,677
Proximal fibula osteotomy (PFO) model	664,971	442,267
Middle fibula osteotomy (MFO) model	664,190	441,591
Distal fibula osteotomy (DFO) model	662,657	440,770

**Table 2:** Material Properties.

	Young’s Modulus (MPa)		Poisson’s Ratio	
Cortical bone [16]	Ex = 6910		Vxy = 0.49	
	Ey = 8510		Vxz = 0.12	
	Ez = 18,400		Vyz = 0.14	
Cancellous bone [17]	1061		0.225	
Cartilage [18]	12		0.45	
Meniscus [18]	80		0.3	
	**MCL** **/LCL** **[19]**	**Proximal Anterior/Posterior Tibiofibula Ligament** **[20]**	**Distal Anterior/Posterior Tibiofibula Ligament** **[21]**	**Anterior/Posterior Interosseous Membrane** **[16,21,22]**
Stiffness (N/mm, per 1 spring)	24/23.2	44.3/36.3	26/33.7	39/39

**Table 3:** Contact conditions.

Contact Body	Contact Type
Cortical-Cancellous bone (Femur, Tibia, Fibula)	Bonded
Femur Bone–Femoral Cartilage	Bonded
Tibia Bone–Tibial Cartilage	Bonded
Meniscus–Tibial Cartilage	Bonded
Femoral Cartilage–Meniscus	Frictional, μ = 0.2
Femoral Cartilage–Tibial Cartilage	Frictional, μ = 0.2
Tibia bone–Fibula Bone	No separation, μ = 0

**Table 4:** Variations in analysis models.

	Bone Model	Intact Model	PFO Model	MFO Model	DFO Model
Force Ratio					
M:L = 2:1		I21	P21	M21	D21
M:L = 3:1		I31	P31	M31	D31
M:L = 4:1		I41	P41	M41	D41

**Table 5:** Peak von Mises stress of the meniscus.

Peak von Mises Stress [MPa]						
Model	M:L = 2:1		M:L = 3:1		M:L = 4:1	
	Medial (vs. Intact)	Lateral (vs. Intact)	Medial (vs. Intact)	Lateral (vs. Intact)	Medial (vs. Intact)	Lateral (vs. Intact)
Intact model	5.000	3.058	5.562	2.574	5.941	2.257
PFO model	4.537 (−9.3%)	2.915 (−4.7%)	4.995 (−10.2%)	2.435 (−5.4%)	5.301 (−10.8%)	2.126 (−5.8%)
MFO model	4.579 (−8.4%)	2.905 (−5.0%)	5.039 (−9.4%)	2.429 (−5.6%)	5.345 (−10.0%)	2.123 (−5.9%)
DFO model	4.579 (−8.4%)	2.911 (−4.8%)	5.040 (−9.4%)	2.433 (−5.5%)	5.346 (−10.0%)	2.125 (−5.9%)

**Table 6:** Peak von Mises stress of the tibial cartilage.

Peak von Mises Stress [MPa]						
Model	M:L = 2:1		M:L = 3:1		M:L = 4:1	
	Medial (vs. Intact)	Lateral (vs. Intact)	Medial (vs. Intact)	Lateral (vs. Intact)	Medial (vs. Intact)	Lateral (vs. Intact)
Intact model	4.681	1.772	5.182	1.503	5.533	1.419
PFO model	3.871 (−17.3%)	1.671 (−5.7%)	4.207 (−18.8%)	1.405 (−6.5%)	4.438 (−19.8%)	1.240 (−12.6%)
MFO model	3.867 (−17.4%)	1.673 (−5.6%)	4.202 (−18.9%)	1.407 (−6.4%)	4.433 (−19.9%)	1.241 (−12.6%)
DFO model	3.867 (−17.4%)	1.668 (−5.8%)	4.202 (−18.9%)	1.404 (−6.6%)	4.433 (−19.9%)	1.240 (−12.6%)

**Table 7:** Peak von Mises stress of the femoral cartilage.

Peak von Mises Stress [MPa]						
Model	M:L = 2:1		M:L = 3:1		M:L = 4:1	
	Medial (vs. Intact)	Lateral (vs. Intact)	Medial (vs. Intact)	Lateral (vs. Intact)	Medial (vs. Intact)	Lateral (vs. Intact)
Intact model	2.124	1.684	2.292	1.366	2.408	1.146
PFO model	1.897 (−10.7%)	1.645 (−2.3%)	2.019 (−11.9%)	1.345 (−1.5%)	2.099 (−12.8%)	1.146 (0.0%)
MFO model	1.897 (−10.7%)	1.645 (−2.3%)	2.019 (−11.9%)	1.346 (−1.5%)	2.099 (−12.8%)	1.147 (0.1%)
DFO model	1.897 (−10.7%)	1.652 (−1.9%)	2.019 (−11.9%)	1.352 (−1.0%)	2.099 (−12.8%)	1.152 (0.5%)

## Data Availability

The datasets used and/or analyzed during the current study available from the corresponding author on reasonable request.
